# A new DNA vaccine expressing HspX-PPE44-EsxV fusion antigens of *Mycobacterium tuberculosis* induced strong immune responses 

**DOI:** 10.22038/ijbms.2020.38521.9171

**Published:** 2020-07

**Authors:** Bagher Moradi, Mojtaba Sankian, Yousef Amini, Aida Gholoobi, Zahra Meshkat

**Affiliations:** 1 Esfarayen Faculty of Medical Sciences, Esfarayen, Iran; 2 Immunology Research Center, Mashhad University of Medical Sciences, Mashhad, Iran; 3 Infectious Diseases and Tropical Medicine Research Center, Resistant Tuberculosis Institute, Zahedan University of Medical Sciences, Zahedan, Iran; 4 Medical Genetics Research Center, Mashhad University of Medical Sciences, Mashhad, Iran; 5 Antimicrobial Resistance Research Center, Mashhad University of Medical Sciences, Mashhad, Iran

**Keywords:** BCG, DNA, Mycobacterium tuberculosis, PCR, Vaccine

## Abstract

**Objective(s):**

Infection with tuberculosis (TB) is regarded as a major health issue. Due to the emergence of antibiotic resistance during TB treatment, prevention via vaccination is one of the most effective ways of controlling the infection. DNA vaccines are developed at a greater pace due to their ability in generating a long-lasting immune response, higher safety compared to the live vaccines, and relatively lower cost of production. In the present study, we evaluated a new DNA vaccine encoding the fusion HspX-PPE44-EsxV antigens, separately, and in combination with Bacillus Calmette–Guérin (BCG) administration, in a prime-boost method in mice.

**Materials and Methods:**

A novel DNA vaccine encoding HspX-PPE44-EsxV fusion antigen of *Mycobacterium tuberculosis* was constructed, and RT-PCR and Western blot analysis were performed to verify the expression of the antigen. Female BALB/c mice were divided into five groups (PBS, BCG, pcDNA3.1 (+) vector, pDNA/HspX-PPE44-EsxV vaccine, and the BCG-prime boost groups). In order to evaluate the immunogenicity of the recombinant vector, BALB/c mice were injected with 100 μg of pDNA at 2-week intervals. Then, cytokine assay was conducted using eBioscience ELISA kits (Ebioscience, AUT) according to manufacturers’ instructions to evaluate the concentrations of IL-4, IL-12, TGF-β, and IFN-γ.

**Results:**

The concentrations of INF-γ, IL-12, and TGF-beta were significantly increased compared to the control groups (*P*<0.001). INF-γ and IL-12 production were increased significantly in pDNA/HspX-PPE44-EsxV+BCG group compared to pDNA/HspX-PPE44-EsxV group (*P*<0.001).

**Conclusion:**

This study showed that the present DNA vaccine could induce a high level of specific cytokines in mice. It was also shown that using this DNA vaccine in a BCG prime-boost protocol can produce significant amounts of IFN-γ, IL-12, and TGF-β.

## Introduction

Tuberculosis (TB), a well-known disease, caused by infection with *Mycobacterium tuberculosis (M. tuberculosis),* has affected human societies at different periods of history (1). Currently, TB is considered a global threat. According to the estimation of the World Health Organization (WHO), one-third of the world’s population is infected with *M. tuberculosis,* which leads to 1,700,000 cases of death annually. Co-infection of TB with human immunodeficiency viruses (HIV) has also increased the TB mortality rate, dramatically (2). Furthermore, the increased rate of infection caused by multiple and extensive-resistant strains to first- and second-line antibiotics has made TB treatment a serious challenge (3). Also, several studies have demonstrated that pregnancy and newborns can be affected by TB (4). Today, the only available vaccine against TB is Bacillus Calmette–Guérin (BCG), which is used subcutaneously in the populations of countries with endemic infection and is also included in the vaccination programs for children. Efficiency of the vaccine against adult pulmonary TB is variable (5). Accordingly, the research, development, and production of a new and more effective TB vaccine are essential (6). Lately, new vaccines such as recombinant BCG, *M. tuberculosis* mutants, DNA vaccines, and subunit vaccines have been produced (7, 8). Among existing vaccine candidates, DNA vaccines are some of the available methods of vaccine design in conferring immunogenicity with viral vectors. These vaccines are expected to provoke the immune system’s responses more effectively compared to the currently available vaccine, BCG (9). DNA vaccines are immunogenic tools that do not cause any infection in recipients. On the other hand, live attenuated vaccines (unlike DNA vaccines) may cause complications in pregnant women and/or people with immune deficiency (10). Antigens such as Hsp60, Hsp70, ESAT–6, PPE44, and HspX are new candidates for vaccine production and/or diagnostic tools of TB (11). It is also noted that simultaneous application of several antigens, compared to the separated forms, leads to a stronger immunity (12). Recently, studies have revealed that PPE44, HspX, and EsxV are immunogenic proteins. HspX protein (α-crystallin) is an antigen expressed only in *M. tuberculosis *(13). This protein is associated with the latent phase of TB infection, and *hspX* gene expression is induced when the oxygen level is low, such environments could, for instance, be found in the host’s macrophage cells. In other words, the expression of this protein causes long-term stability of this bacteria in the host’s macrophage cells (14). Previous studies have shown that vaccine candidates containing the HspX antigen can induce an immunogenic and protective efficacy even more than BCG vaccine (15-17). Another immunodominant mycobacterial antigen is PPE44. This antigen is expressed in *M. tuberculosis* and belongs to the PPE protein family (Pro–Pro–Glu). Recent studies have proposed PPE44 as a new and powerful candidate for vaccine production. This antigen has specific epitopes, exclusively detectable by major histocompatibility complex I (MHC–I) and can induce desirable protection when used as subunit or DNA vaccines (18, 19). EsxV is another immunogenic antigen and is an important protein in the secretion of PE/PPE family proteins (20). Villarreal *et al*. (2014), indicated immunogenicity of 15 antigens of the EsxV family. In this study, evaluation of cellular immune response, including assessment of interferon gamma (IFN–γ) production, as an important activator of macrophages and T CD 8+ cells was performed (21). CD8+ T-cell-dependent protective immunity and production of higher IFN-γ can be induced by mycobacterial antigens such as PPE44 and EsxV that contain MHC-1 restricted epitopes (18, 21). In fact, induction of CD8+ T-cell and production of IFN-γ is an effective response to *M. tuberculosis* and other intracellular bacteria. Consequently, this antigen can be used as a specific antigen in IFN-γ production.

The prime-boost method is an approach that has been studied in previous efforts. In this strategy, BCG vaccination is followed by a DNA vaccine injection containing highly immunogenic antigens. This approach activates CD8+ T cell immune responses strongly, compared to BCG vaccine (22). So, in the present study, we constructed a novel DNA vaccine containing Hspx–PPE44–EsxV fusion gene and evaluated its efficacy in the stimulation of immune responses, separately, and in combination with BCG in the animal model. 

## Materials and Methods

***Animal model***

Female BALB/c (six- to eight-weeks-old) mice were purchased from Razi Institute (Mashhad, Iran). Mice were divided into five groups (phosphate-buffered saline (PBS) group, BCG group, pcDNA3.1 (+) vector group, pDNA/HspX-PPE44-EsxV vaccine group, and the BCG-prime boost group). Six mice in each group were included and maintained under standard conditions (23, 24). 

***Plasmid construction***

Design of fusion gene, cloning and construction of the recombinant pcDNA3.1 (+), colony-PCR, and restriction enzyme analysis were performed (25). Optimized HspX-PPE44-EsxV fusion segment was synthesized at the Generay Company (China). Then, the HspX-PPE44-EsxV fusion segment was cloned into pcDNA3.1 (+). We confirmed the accuracy of cloning by restriction enzyme analysis and sequencing of the fusion construct. 

***In vitro expression of recombinant pcDNA3.1 (+)***

In order to transfect recombinant pcDNA3.1 (+), Chinese hamster ovary (CHO) cells seeded into 6-well micro-plates were incubated overnight. Transfection was conducted in 80% confluency of cell culture. CHO cells were transfected with 1 μg of recombinant pDNA3.1 (+)/PPE44-EsxV-HspX plasmid in 2 μl of Escort-IV transfection reagent according to the manufacturer’s instructions (Sigma Aldrich, USA). After 48 hr, the cells were collected using 0.5 ml trypsin (Invitrogen, USA). Then, total RNA was extracted using RNA extraction kit according to the manufacturer’s instructions (Pars-Toos, Iran). Purified RNA was added to the final mixture and was synthesized using a cDNA synthesis kit (Pars Tous, Iran). Then, cDNA was amplified by PCR. Expression of the HspX-PPE44-EsxV fusion protein was confirmed by Western blot analysis using mouse anti-His tag primary antibody. Also, peroxidase-conjugated rabbit anti-mouse IgG was used as a secondary antibody (AbD SeroTec, USA). 

***Vaccination***

At first, mice in BCG and BCG prime-boost groups were immunized subcutaneously with BCG vaccine, a live attenuated strain of *Mycobacterium bovis* (5×10/ CFU, Strain of Paris Pasteur Institute)(26). After two weeks, 100 μg of the recombinant vaccine was injected intramuscularly to all mice except PBS and vector groups (24). Immediately after injection, electroporation was conducted in order to stimulate the muscle to uptake higher volumes of the DNA vaccine. The vaccination sites of mice were electroporated at 200 volts/cm with electrodes distanced 2 to 5 mm and six pulses of 20 ms each (27). In the vector group, 100 μg of pCDNA3.1 (+) vector was injected (16). These injections were performed three times after the first injection of BCG, and at 2-week intervals. Finally, the mice were sacrificed after three weeks of the last injection.

***Cytokine assay***

The spleens of sacrificed mice were removed aseptically, and splenocytes purification was performed using the lysis buffer. Leukocytes were collected after counting the number of living cells (2×cells/ml). The cells were cultured in duplicate in RPMI 1640 with 10% fetal bovine serum (Bioidea, Iran) and 1% penicillin/streptomycin (PenStrep100x, Biosera, UK), in the presence of 5 μg/ml of HspX -PPE44 -EsxV fusion antigen (28), at 37 ^°^C with 5% CO_2_ for 72 hr. In our study, positive control cultures were stimulated by phytohemagglutinin-A (PHA) (2 μg/μl: concentration of PHA), (Sigma Aldrich, united states) while our negative cultures were not stimulated. After 72 hr, cell culture supernatants were harvested and stored in -80 ^°^C for the subsequent cytokines assay.

Cytokine responses were measured using eBioscience ELISA kits (Ebioscience, AUT) according to its instruction to evaluate the concentrations of interleukin-4 (IL-4), IL-12, transforming growth factor β (TGF-β), and IFN-γ cytokines produced in the cell culture. 

***Statistical analysis***

SPSS 16.0 (IBM, Armonk, NY) was used for performing the statistical analysis. Two way ANOVA test was used to compare the means. In all statistical tests, the significance level of *P*<0.001 was considered.

## Results

***RT-PCR and Western blot analysis***

**Figure 1:**
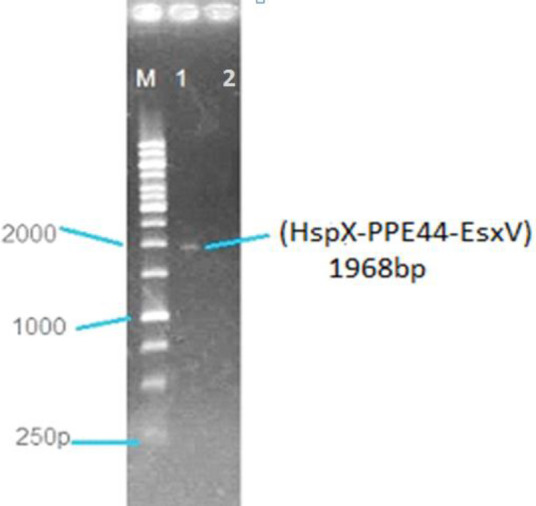
RT-PCR results on cDNA from transfected Chinese hamster ovary (CHO) cells (lane number 1) and non-transfected CHO cells (lane number 2); M: 1 kb DNA size marker (Fermentas, Germany)

**Figure 2:**
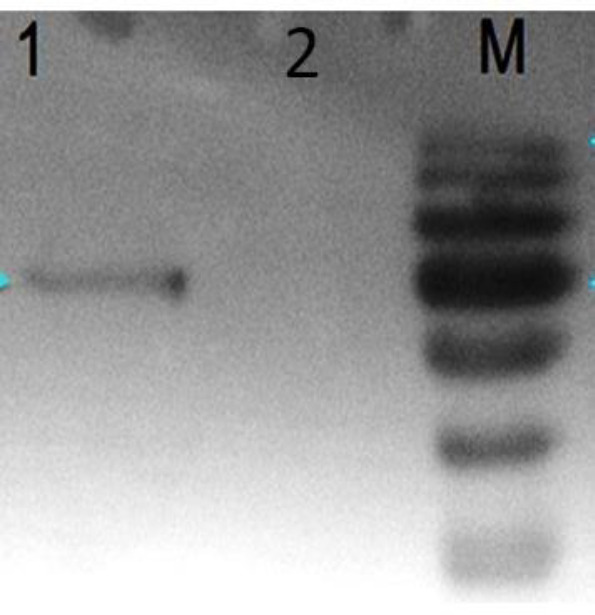
Western blot analysis of HspX-PPE44-EsxV transfected Chinese hamster ovary (CHO) cells (lane 1) and untransfected CHO cells (lane 2) and lane m: protein size marker (Fermentas Company, Germany)

To confirm HspX-PPE44-EsxV expression, RT-PCR and Western blot were performed. RT-PCR was performed on RNA isolated from the transfected cells, and as we expected, a 1968 bp band was identified by Agarose gel electrophoresis (Figure 1). To evaluate HspX-PPE44-EsxV protein production, Western blots were conducted. As we expected, a 68 kDa band was detected by Western blot using rabbit anti-mouse IgG antibody (AbD Serotec, UK) (Figure 2).

***Cytokine assay***

**Figure 3:**
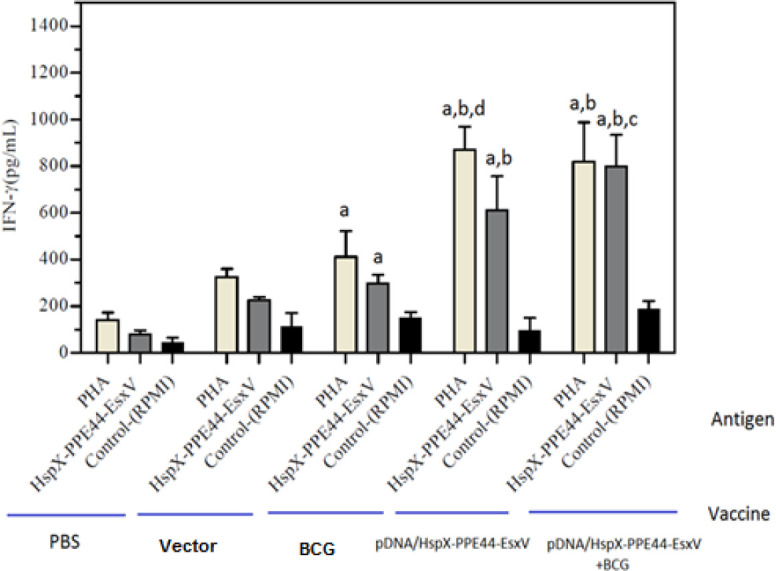
IFN-γ responses in the spleen cells of vaccinated mice in pDNA/HspX-PPE44-EsxV group, pDNA/HspX-PPE44-EsxV+BCG and control group (BCG, PBS and / or Vector). (a) *P*<0.001 compared to control group (PBS and/or Vector). (b) *P*<0.001 compared to positive and negative control groups (PBS, Vector, BCG). (c) *P* <0.001 compared to groups receiving vaccine pDNA/HspX-PPE44-EsxV (DNA Vaccine Group). (d) *P*<0.001 compared to groups receiving vaccine of pDNA/HspX-PPE44-EsxV / BCG (Prime - boost Group). IFN–γ: Interferon gamma, BCG: Bacillus Calmette–Guérin

**Figure 4:**
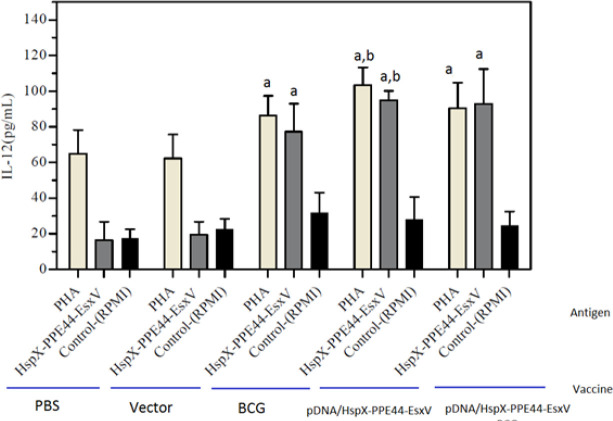
IL-12 responses in the spleen cells of vaccinated mice in pDNA/HspX-PPE44-EsxV group, pDNA/HspX-PPE44-EsxV +BCG and control group (BCG, PBS and/or Vector). (a) *P*<0.001 compared to control group (PBS and/or Vector). (b) *P*<0.001 compared to positive and negative control

The concentrations of IFN-ᵧ, IL-12, TGF-beta, and IL-4 were measured to evaluate immune responses. IFN-γ levels were significantly increased in all groups of mice, and there was a significant difference in IFN-γ production between the DNA vaccine group (pDNA/HspX-PPE44-EsxV group) (612.25±144.32) and BCG prime-boost group (pDNA/HspX-PPE44-EsxV+BCG group) (799.11±135.51) with the BCG group (297.43±37.48). The increased level of IFN-γ implied the strong immune response of the DNA vaccine group (*P<*0*.*001) (Figure 3). Evaluation of IL-12 concentration showed significant difference in pDNA/HspX-PPE44-EsxV (94.90±5.28), pDNA/HspX-PPE44-EsxV+BCG (92.88±19.50), and BCG groups (77.25±15.86) compared to vector (19.46±7.31) and PBS (16.48±10.39) groups (*P*<0.001) (Figure 4). 

**Figure 5:**
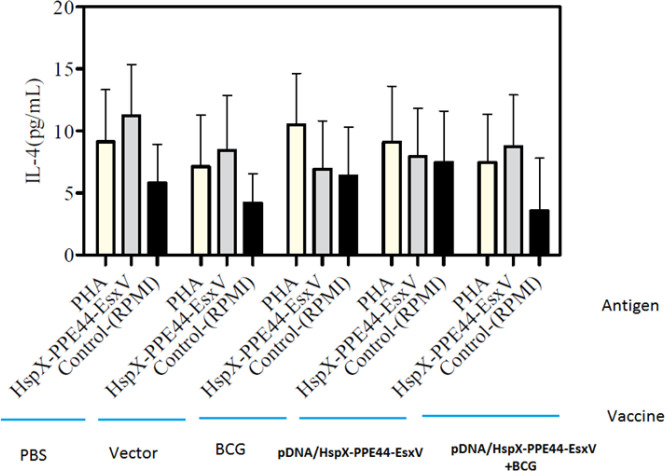
IL-4 responses in the spleen cells of vaccinated mice in pDNA/HspX-PPE44-EsxV group, pDNA/HspX-PPE44-EsxV+BCG and control group (BCG, PBS and / or Vector). (a) *P*<0.001 compared to control group (PBS and/or Vector). (b) *P *<0.001 compared to positive and negative control groups (PBS, Vector, BCG). (c) *P*<0.001 compared to vaccine receiving groups (Prime-boost group). BCG: Bacillus Calmette–Guérin, IL-4: Interleukin-4

**Figure 6:**
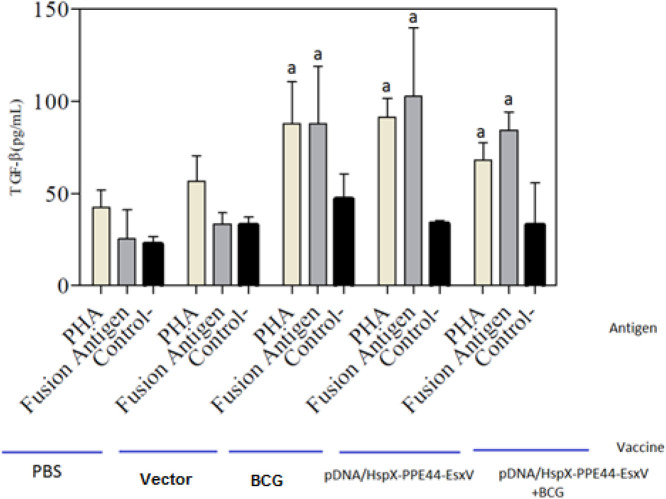
TGF-β production by spleen cells of vaccinated mice in pDNA/HspX-PPE44-EsxV group, pDNA/HspX-PPE44-EsxV+BCG and control group (BCG, PBS and / or Vector). (a) *P*<0.001 compared to control group (PBS and / or Vector). (b) P <0.001 compared to positive and negative control groups (PBS, Vector, BCG). (c) *P*<0.001 compared to P/HspX-PPE44-EsxV/BCG vaccine receiving groups (Prime-boost group). TGF-β: Transforming growth factor β, BCG: Bacillus Calmette–Guérin

There was no significant difference in IL-4 production between vaccinated groups and control groups (Figure 5). An important marker of Treg, TGF-beta, was higher in all groups compared to the vector and PBS groups. Besides, TGF-beta production showed significant difference in pDNA/HspX-PPE44-EsxV (102.61±34.20), pDNA/HspX-PPE44-EsxV+BCG (84.23±9.93), and BCG groups (87.71±31.16) compared to vector (33.35±6.10) and PBS (25.28±15.77) groups (*P*<0.001). No significant differences were observed between BCG and DNA vaccinated groups (Figure 6).

**Figure 7:**
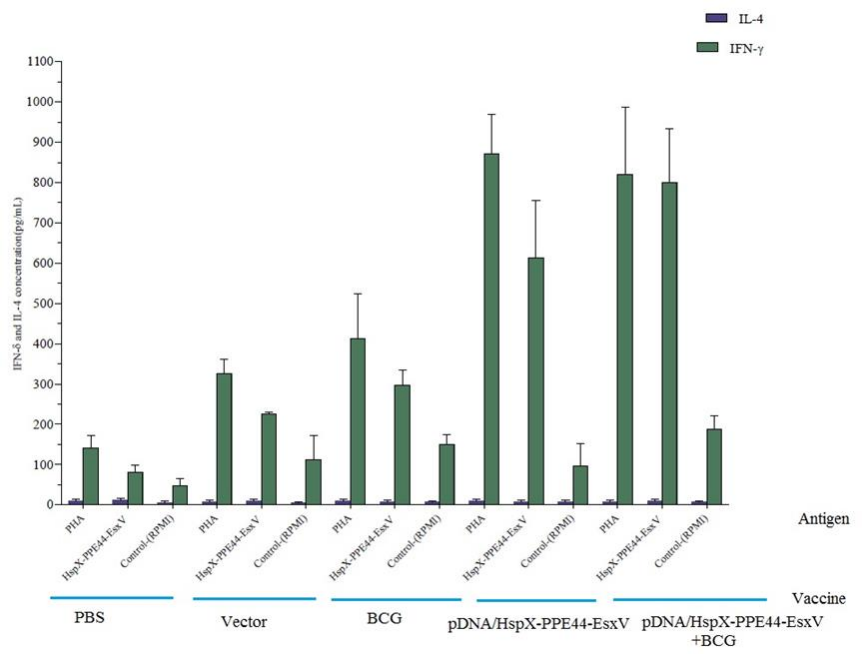
Comparison of IL-4 and IFN-γ levels of vaccinated mice in pDNA/HspX-PPE44-EsxV group, pDNA/HspX-PPE44-EsxV+BCG and control group (BCG, PBS and / or Vector) (pg/ml). BCG: Bacillus Calmette–Guérin, IL-4: Interleukin-4, IFN–γ: Interferon gamma

Comparison of IL-4 and IFN-γ concentration showed that Th1 was activated in all groups of mice (Figure 7).

## Discussion

Currently, the BCG vaccine is the only available vaccine against TB, which has variable immunity against pulmonary TB in different populations (29). Vaccine candidates should have at least one of the two properties recommended by the WHO: 1) should be more effective compared to BCG; 2) could improve the efficiency of the BCG vaccine in a prime-boost method. Extensive studies were conducted to develop an effective and safe vaccine against TB. A relatively new strategy is to produce DNA vaccines to improve the efficiency of the current vaccine. In this strategy, antigens of *M. tuberculosis* that are expressed less or not expressed in BCG are produced and used as DNA vaccines in a prime-boost method, in which a dose of BCG is injected in mice and after the interval time elapsed, other doses of DNA vaccine can be injected to mice (30, 31). In our study, a new DNA vaccine was injected to mice groups alone and in a BCG prime-boost regimen. Another study, showed that DNA vaccine encoding hspX could induce immune responses in the prime-boost strategy (32). This finding revealed that the DNA vaccine inducing hspX antigen can be more efficient along with the BCG vaccination. One of the main areas of TB research is the detection of more effective antigens, which stimulate cellular and humoral immunity (33). Recently, antigens expressed in the delayed phase of TB infection, such as Rv2031c (HspX) have attracted attention (34). This protein is expressed only in *M. tuberculosis* and can stimulate the immune responses (35). Application of pDNA**-**HspX-PPE44-EsxV vaccine was safe, and no intolerance was observed in the treated animals. In the present study, pDNA**-**HspX-PPE44-EsxV was administered without any adjuvant and based on the results, it seems that this fusion DNA vaccine has the ability to stimulate immune responses in mice model.

 In Abdallah *et al. *(2008) study, a recombinant Bacille Calmette–Guérin (rBCG) expressing HspX was injected to an animal model using prime-boost vaccination (20). These results were in accordance with ours, showing induced cellular immune responses. In our study, TGF–ß concentration is also considered as a marker of T–reg cells and proliferation and differentiation of Th17 cells, which increased in vaccinated groups compared to negative control group, suggesting that DNA vaccine encoding HspX–PPE44–EsxV could adjust the cellular and humoral immune responses (36). TGF-β and IL-12 are necessary for the maintenance of T cells responses and are important in the prime-boost regimen. Also, our study showed that pDNA-HspX-PPE44-EsxV can produce TGF-β and IL-12 and could be used in the heterologous prime-boost method.

*M. tuberculosis* is an intracellular pathogen, which can be controlled by cellular immunity and IFN–γ secreted by TCD8 cells (37, 38). During TB infection, the levels of IFN–γ, IL–12, and tumor necrosis factor alpha (TNF–α) cytokines show activation of macrophages and cellular immune response. It is known that the production of IL–12 is important in IFN–γ secretion, and activation of dendritic cells can provide protection against tuberculosis (38). Then, the dendritic cells expose the processed mycobacterial antigens to CD8+ cells and induce more effective immune responses (39, 40). So, an effective vaccine against TB must have three immunogenic properties: 1) high Th1/Th2 response, 2) induction of more durable responses, and 3) balanced cellular and humoral immune response or Th1/Th2 responses. In the present study, IL–12 and IFN–γ prime production can confirm activation of effective immune responses. Also, Romano *et al.* and Bruffaerts *et al.* showed the immunogenicity and protective properties of a DNA vaccine encoding PPE44 antigen in infected mice (18, 19). Moreover, in several studies, we demonstrated that HspX-Ppe44-EsxV subunit vaccine alone and with a combination of nanoparticles represents a large immunological potential to induce cellular immune responses (13, 28). Similar to previous studies, in our study, the immunogenicity of the DNA vaccine was confirmed. The production of IFN-γ and IL–12 in all groups of mice was increased, and there was a significant difference in IFN-γ and IL–12 production between pDNA/HspX-PPE44-EsxV and pDNA/HspX-PPE44-EsxV+BCG groups with BCG, vector, and PBS groups. In Villarreal *et al.* study (2014) on the immunogenicity of 15 antigens of EsxV family, five types of DNA vaccine were produced. In this study, evaluation of cellular immune response, including assessment of IFN–γ production, as an important activator of macrophages, T CD4+ and 8+ cells were performed (21). These results partly correspond with the results of our study, such that the levels of IFN–γ in spleen cell cultivation of DNA-vaccinated and prime-boost groups were significantly increased compared to BCG, vector, and PBS groups. Moreover, assessment of cytokines that are involved in the induction of humoral immune response, such as IL–4 and TGF–β could be considered. These cytokines adjust cellular immune response at appropriate concentrations and pave the way for humoral immune response at higher concentrations. TGF–β is produced by Treg cells that decrease the activation of macrophages by their anti-inflammatory properties and produce oxygen and nitrogen radicals and adjust cellular immune response to humoral immunity (41). Results of previous studies show that in our study, TGF–β was elevated compared to the control group. Th2 cells can adjust immune response by the production of IL–4 and increasing the production of antibodies (42). 

Moreover, Yuan *et al.* (2012) designed a DNA vaccine encoding HspX antigen and evaluated its immunity similar to our study. Increased IFN–γ showed activated cellular immune response in the DNA-vaccinated group (34). In the present study, comparison of concentrations of IL–4, 12, and IFN–γ in all groups showed that cellular immune responses and Th1 lymphocytes were more activated. Also, comparison of TGF–ß concentrations in all groups of this study was in accordance with the results of other studies. So, our DNA vaccine can balance Th1 and Th2 responses, which are some of the major properties of TB vaccine candidates. Further studies are required to evaluate the protective efficiency of this DNA vaccine on infected mice in order to decrease the bacterial load in mice lungs. 

**Table 1:** Cytokine responses in the spleen of vaccinated BALB/c mice

TGF-β	IL-4	IL-12	IFN-γ	Cytokine
				Groups
25.28±15.77	14.53±5.43	16.48±10.39	80.45±16.77	Control
87.71±31.16	16.93±6.30	77.25±15.86	297.43±37.48	BCG
102.61±34.20	27.11±3.66	94.90±5.28	612.25±144.32	DNA vaccine
84.23±9.93	97.55±19.10	92.88±19.50	799.11±135.51	DNA vaccine +BCG

Six mice in every group were sacrificed after 3 weeks of the last HspX -PPE44 -EsxV injection. The splenocytes were cultured, and BCG and fusion antigens were added to culture. Cytokine assay were performed after 72 hr on splenocytes supernatant. Values are expressed in pg/ml and represented as mean± standard deviation. The mice were vaccinated in prime-boost method and the results were analyzed by two-way ANOVA test, and significant difference was set as *P*<0.001

groups (PBS, Vector, BCG). BCG: Bacillus Calmette–Guérin, IL-12: Interleukin-12

## Conclusion

Cellular immune responses are critical in TB controlling. Cytokines such as IFN-γ and IL-12 are important Th1 markers included in the cell-mediated immunity. In the present study, the levels of IFN-γ, IL-12 and TGF-β were elevated. These findings indicate that this construct can stimulate cell mediate immunity strongly. Results of this study indicate that this vaccine can be considered as a construct that can induce Th1 responses, especially in prime-boost strategy.
